# Clinicopathologic characteristics of ductal carcinoma in situ and risk of subsequent invasive breast cancer: a multicenter, population-based cohort study

**DOI:** 10.1007/s10549-024-07599-x

**Published:** 2025-01-20

**Authors:** Thomas E. Rohan, Yihong Wang, Fergus Couch, Heather Spencer Feigelson, Robert T. Greenlee, Stacey Honda, Azadeh Stark, Dhananjay Chitale, Chenxin Zhang, Xiaonan Xue, Mindy Ginsberg, Olivier Loudig

**Affiliations:** 1 Department of Epidemiology and Population Health, Albert Einstein College of Medicine, Bronx, NY USA; 2 Department of Pathology and Laboratory Medicine, Rhode Island Hospital and Lifespan Medical Center, Warren Alpert Medical School of Brown University, Providence, RI USA; 3 Department of Laboratory Medicine and Pathology, Mayo Clinic, Rochester, MN USA; 4 Department of Health Sciences Research, Mayo Clinic, Rochester, MN USA; 5 Institute for Health Research, Kaiser Permanente, Aurora, CO USA; 6 Center for Clinical Epidemiology and Population Health, Marshfield Clinic Research Institute, Marshfield, WI USA; 7 Center for Integrated Healthcare, Kaiser Permanente, Hawaii Permanente Medical Group, Honolulu, HI USA; 8 Department of Pathology and Laboratory Medicine, Henry Ford Health System, Detroit, MI USA; 9 Breast Oncology Program and Department of Pathology, Henry Ford Health System, Detroit, MI USA; 10 Center for Discovery and Innovation (CDI), Hackensack Meridian Health, Nutley, NJ USA

**Keywords:** Ductal carcinoma in situ, Clinicopathologic, Risk factors, Invasive breast cancer

## Abstract

**Purpose:**

To study the association between clinicopathologic characteristics of ductal carcinoma in situ (DCIS) and risk of subsequent invasive breast cancer (IBC).

**Methods:**

We conducted a case–control study nested in a multicenter, population-based cohort of 8175 women aged ≥ 18 years with DCIS diagnosed between 1987 and 2016 and followed for a median duration of 83 months. Cases (n = 497) were women with a first diagnosis of DCIS who developed a subsequent IBC ≥ 6 months later; controls (2/case; n = 959) were matched to cases on age at and calendar year of DCIS diagnosis. Univariable and multivariable conditional logistic regression models were used to examine the associations between the DCIS characteristics of interest (non-screen detection of DCIS, tumor size, positive margins, grade of DCIS, necrosis, architectural pattern, microcalcification, and estrogen receptor (ER), progesterone receptor (PR), and human epidermal growth factor receptor 2 (HER2) status) and risk of IBC.

**Results:**

In the total study population, the associations were largely null. In subgroup analyses, there were strong position associations with punctate necrosis (pre/perimenopausal women), detection by physical exam (postmenopausal women), architectural patterns other than the main types (breast-conserving surgery [BCS]), and DCIS margins (ipsilateral cases), and inverse associations with HER2 positivity (BCS) and microcalcification (mastectomy); however, the associated confidence intervals were mostly very wide.

**Conclusion:**

The results of this study provide limited support for associations of the DCIS clinicopathologic characteristics studied here and risk of IBC.

**Supplementary Information:**

The online version contains supplementary material available at 10.1007/s10549-024-07599-x.

## Introduction

Ductal carcinoma in situ (DCIS) of the breast, a neoplastic proliferation of mammary ductal epithelial cells confined to the ductal-lobular system [1], is considered to be a non-obligate precursor of invasive breast cancer (IBC) [2]. DCIS lesions exhibit heterogeneous biologic behavior, with some being rapidly progressive and others progressing more slowly or not at all [1].

Treatment of DCIS is focused on preventing its recurrence or progression to IBC. However, treatment approaches vary, and many DCIS patients are either undertreated or overtreated [3, 4]. Characterization of DCIS lesions that are associated with increased risk of IBC development may help to tailor treatment for individual patients, thereby reducing the risk of overtreatment for those with relatively low risk of IBC [5, 6]. In this regard, a number of clinicopathologic characteristics of DCIS have been related to the risk of IBC following DCIS. Potentially relevant variables, as summarized in several meta-analyses and pooled analyses, include non-screen detection of DCIS (e.g., by palpation), tumor size, positive margins, grade of DCIS, comedonecrosis, architectural pattern, microcalcification, and receptor status [estrogen receptor (ER), progesterone receptor (PR), and human epidermal growth factor receptor 2 (HER2)] [7–12]. However, the results of studies to date have not been entirely consistent, due to factors such as inconsistent definition of predictor variables, simultaneous inclusion in some studies of women with invasion or microinvasion, relatively small sample sizes, and also to insufficient measurement of and adjustment for confounding variables [8]. These considerations highlight the need for independent validation of risk factors identified to date [8]. Also, although most studies have focused on ipsilateral IBC recurrence, it is of interest to determine whether these factors are also associated with contralateral recurrence given that molecular (and associated histological) changes occur in the tissue of both breasts in response to exposure to endogenous and environmental factors [13].

In the large, population-based study reported here, we investigated the associations between DCIS clinicopathologic characteristics and risk of subsequent IBC.

## Methods

### Study population and design

The study protocol has been described in detail elsewhere [13]. In brief, we established a multi-center population-based cohort of 8175 women aged ≥ 18 years, with no history of IBC, who received their first histologic diagnosis of DCIS between January 12, 1987, and December 20, 2016 (women with microinvasion were excluded). The cohort consists of DCIS patients from six large community-based integrated health care delivery systems across the United States: Henry Ford Health (HFH) (Detroit, MI), Kaiser Permanente (KP) Colorado (Denver, CO), KP Hawaii (Honolulu, HI), Marshfield Clinic (Marshfield, WI), Mayo Clinic (Rochester, MN), and Montefiore Medical Center (Bronx, NY).

### Cohort follow-up and ascertainment of IBC cases

We used the electronic medical record (EMR) and administrative databases at each institution to follow patients passively from the date of their DCIS diagnosis until the date of development of subsequent IBC, last contact, health plan disenrollment, or death, whichever came first. The occurrence of subsequent IBC (ipsilateral or contralateral) was ascertained in each center by record linkage to the respective tumor registry and/or to other EMR data. Given that all participating centers provide integrated care, all health care procedures for patients are typically conducted within the health systems, and any outside services are captured through reimbursement/claims data.

### Nested case–control study

The present investigation was conducted as a case–control study nested within the DCIS cohort. Cases were women with a first diagnosis of DCIS and with a subsequent diagnosis of IBC at least 6 months after the index DCIS diagnosis. Controls were women with a first diagnosis of DCIS, with no history of bilateral mastectomy prior to the date of diagnosis of IBC for the corresponding case, and who were alive but had not developed IBC during the same follow-up period as that for the corresponding case. For each of the women with DCIS who developed a subsequent IBC (cases), we selected two controls using risk-set sampling. Each control was individually matched to the corresponding case on calendar year of (mostly within ± 1 year) and age at (generally ± 1 year) diagnosis of DCIS. A total of 497 cases and 959 controls were included in the study. The numbers of cases and controls (respectively) included from each center were as follows: HFH, 147, 289; KP Colorado, 33, 63; KP Hawaii, 67, 130; Marshfield Clinic, 68, 133; Mayo Clinic, 110, 220; and Montefiore Medical Center, 72, 124.

### Risk factor data

We used the electronic medical records at each participating institution to extract data on variables related to the patient (e.g., age at and year of DCIS diagnosis), the DCIS lesion (e.g., method of detection, lesion size) and DCIS treatment (e.g., breast conserving treatment, radiotherapy). The data were obtained in a standardized manner across institutions by using a chart abstraction manual to guide data abstraction; chart abstractors were trained centrally in use of the manual. Risk factor data were abstracted for all cases and their matched controls, regardless of whether DCIS tissue was obtained.

### Tissue blocks/sections

For the cases and matched controls, we attempted to retrieve formalin-fixed paraffin-embedded DCIS tissue blocks from the pathology archives of the participating institutions. We obtained tissue blocks/sections for 322 (64.8%) cases and 600 (62.6%) controls.

### Histopathology/immunostaining

Hematoxylin & eosin-stained sections were reviewed and classified according to standard criteria with respect to nuclear grade, architectural pattern, necrosis, and microcalcification [14]. Additional sections were stained for ER, PR, and HER2, and interpreted as per standard surgical pathology practice in accordance with ASCO-CAP guidelines [15, 16]. ER/PR positivity was defined as ≥ 1% cells staining positive [15] and HER2 positivity was defined as a score of 3+ [16].

All reviews were performed without knowledge of patient data, treatment, or outcome (case–control status). For those women for whom tissue blocks were not located (n = 534), we used information on tumor characteristics, histology, and receptor status abstracted from the medical records.

### Statistical analysis

Baseline characteristics of the study participants were summarized by case–control status using means [standard deviations (SD)] or medians [interquartile ranges (IQR)] for continuous variables, and frequencies and percentages of total (%) for categorical variables.

Conditional logistic regression models were used to estimate odds ratios (OR) and 95% confidence intervals (CI) for the associations between the clinicopathologic characteristics of interest and risk of subsequent IBC (ipsilateral or contralateral). Univariable and multivariable conditional logistic regression analyses were conducted separately for the following clinicopathologic variables: method of DCIS detection, lesion size, DCIS margin, DCIS nuclear grade, DCIS architectural pattern, necrosis, microcalcification, ER, PR, and HER2. The multivariable logistic analyses were adjusted for the following potential confounders: type of DCIS treatment, race, family history of breast cancer, age at menarche (years), age at first live birth (years), history of bilateral oophorectomy, menopausal status, use of hormone replacement therapy (HRT), and body mass index (BMI)(kg/m^2^). In further analysis, we examined all clinicopathologic variables of interest plus the confounders simultaneously, followed by a backward variable selection process to determine a final model.

A sensitivity analysis was conducted using multiple imputation on variables with missing rates below 20% [17, 18]. The variables imputed (with their respective missing rates) were: lesion size (12.84%), use of HRT (10.23%), type of DCIS treatment (9.13%), race (8.10%), DCIS nuclear grade (6.18%), history of bilateral oophorectomy (4.26%), BMI (4.26%), family history of breast cancer (3.43%), and menopausal status (1.79%). A multivariable model was then run for each of the clinicopathologic variables using the imputed dataset.

To examine if the associations of the clinicopathologic variables with risk of breast cancer development varied over time, multivariable conditional logistic regression models with interaction terms between the primary DCIS variables and time to diagnosis of IBC were fitted.

Using multivariable conditional logistic regression, the associations of the clinicopathologic variables with risk of IBC were examined further in ipsilateral cases and matched controls (number of records = 373), in contralateral cases and matched controls (n = 457), and, because the extent of missingness was higher in the earlier part of the DCIS diagnosis eligibility period, in women whose DCIS was diagnosed between 1987 and 2001, and separately, in women whose DCIS was diagnosed between 2002 and 2016. Furthermore, after breaking the matching, the associations were also examined in women for whom DCIS tissue was received (n = 922), in White women (n = 902), in Black women (n = 267), in pre/perimenopausal women (n = 357), in postmenopausal women (n = 1073), in women who received breast-conserving surgery (BCS) only (n = 235), in women who received BCS and radiation therapy or BCS with radiation and hormone therapy (n = 682), and in women who underwent mastectomy (n = 332); these analyses were performed using multivariable unconditional logistic regression, with adjustment for the confounders (see earlier) and additional adjustment for the matching variables (age at and year of DCIS diagnosis).

The statistical analyses were performed using R version 4.3.1 [19]. All statistical tests were two-sided, with statistical significance indicated by *p* < 0.05.

## Results

During a median (interquartile range) follow-up period of 83 (46, 130) months, 497 incident cases of IBC were ascertained. Table 1 summarizes the baseline characteristics of the cases and controls. Cases and controls were closely matched on age at and calendar year of DCIS diagnosis (as per the study design). Compared to controls, cases were more likely to have been treated with BCS or BCS plus radiotherapy, to be Black, to have a relatively high BMI, to have a family history of breast cancer, to have a relatively late age at menarche, and to have had a first live birth at a relatively young age.

**Table 1:** Baseline characteristics of the study subjects

Characteristic	Cases (n = 497)	Controls (n = 959)
Age at DCIS diagnosis, y (mean, SD)	60.0 (12.4)	60.0 (12.1)
Calendar year of DCIS diagnosis (median, 25th, 75th percentile)	2004 (1998, 2009)	2004 (1998, 2009)
Race, n (%)		
White	290 (58.4)	612 (63.8)
Asian	44 (8.9)	71 (7.4)
Black	109 (21.9)	158 (16.5)
Other racial group or multiracial	20 (4.0)	34 (3.5)
Missing	34 (6.8)	84 (8.8)
Method of DCIS detection, n (%)		
Mammogram	398 (80.1)	801 (83.5)
Physical exam	63 (12.7)	100 (10.4)
Ultrasound/MRI/Molecular imaging	1 (0.2)	5 (0.5)
Missing	35 (7.0)	53 (5.5)
DCIS treatment, n (%)		
Breast conserving surgery (BCS)	97 (19.5)	138 (14.4)
BCS + Radiotherapy (RT)	151 (30.4)	233 (24.3)
BCS + RT + Hormone therapy (HT)	77 (15.5)	221 (23.0)
Other (RT, HT, BCS + HT)	32 (6.4)	52 (5.4)
Mastectomy	101 (20.3)	237 (24.7)
Missing	39 (7.8)	78 (8.1)
Menopausal status, n (%)		
Premenopausal	97 (19.5)	173 (18.0)
Perimenopausal	25 (5.0)	62 (6.5)
Postmenopausal	368 (74.0)	705 (73.5)
Missing	7 (1.4)	19 (2.0)
Body mass index (kg/m^2^), n (%)		
< 25	133 (26.8)	265 (27.6)
25–29.9	138 (27.8)	300 (31.3)
30–34.9	112 (22.5)	201 (21.0)
≥ 35	95 (19.1)	150 (15.6)
Missing	19 (3.8)	43 (4.5)
Family history of breast cancer, n (%)		
No	339 (68.2)	712 (74.2)
Yes	146 (29.4)	209 (21.8)
Missing	12 (2.4)	38 (4.0)
Age at menarche (years), n (%)		
< 12	56 (11.3)	93 (9.7)
≥ 12	336 (67.6)	603 (62.9)
Missing	105 (21.1)	263 (27.4)
Age at first live birth (years), n (%)		
Never/Not completed pregnancy	63 (12.7)	118 (12.3)
< 30	287 (57.7)	500 (52.1)
> = 30	48 (9.7)	100 (10.4)
Missing	99 (19.9)	241 (25.1)

The associations of clinicopathologic characteristics with risk of IBC overall are shown in Tables 2 and 3. There were small increases in the risk of IBC for those whose DCIS was detected by physical exam, for those with a cribriform DCIS architectural pattern, and for those with punctate necrosis (Table 2), and there were small decreases in risk for those who were ER-negative, PR-negative, or HER2 positive (Table 3). However, none of these associations was statistically significant. The associations were mostly unchanged after we performed imputation for those variables (exposures and potential confounders) that had missing rates of less than 20% (Table 2), although the associations for nuclear grade, punctate necrosis, and HER2 positivity were closer to the null, while there was a slight (statistically non-significant) increase in risk of IBC for those who were ER negative (Table 3).There was a strong positive association between involved DCIS margins and risk of ipsilateral IBC, albeit with very wide confidence intervals (OR = 6.02, 95% CI 1.25–29.12) (Table 4). Associations for contralateral cases were mostly in the opposite direction to those for ipsilateral cases but were statistically non-significant, with confidence intervals that overlapped those for the corresponding associations for ipsilateral IBC (Table 5).

**Table 2:** Associations of clinicopathologic characteristics of DCIS with risk of subsequent invasive breast cancer

Characteristic	Cases (n = 497)	Controls (n = 959)	Univariable OR (95% CI)	Multivariable OR (95% CI)	Multivariable OR (95% CI) with imputation
Method of DCIS detection					
Mammogram	398 (80.1)	801 (83.5)	1^b^	1^b^	1^b^
Physical exam	63 (12.7)	100 (10.4)	1.26 (0.89, 1.79)	1.19 (0.77, 1.84)	1.23 (0.85, 1.77)
Ultrasound/MRI/Molecular imaging/missing^a^	36 (7.2)	58 (6.0)	1.20 (0.72, 2.01)	1.35 (0.59, 3.10)	1.33 (0.77, 2.31)
Lesion size (mm)					
< = 8	152 (30.6)	256 (26.7)	1^b^	1^b^	
8–20	145 (29.2)	290 (30.2)	0.84 (0.63, 1.12)	0.76 (0.53, 1.10)	0.87 (0.62, 1.21)
> = 20	138 (27.8)	288 (30.0)	0.83 (0.62, 1.10)	0.77 (0.54, 1.12)	0.88 (0.64, 1.21)
Missing	62 (12.5)	125 (13.0)	0.85 (0.57, 1.26)	0.73 (0.40, 1.33)	–
DCIS margin					
Clear (≥ 2 mm)	121 (24.3)	206 (21.5)	1^b^	1^b^	1^b^
Involved (< 2 mm)	82 (16.5)	163 (17.0)	0.86 (0.61, 1.22)	0.99 (0.63, 1.57)	0.87 (0.61, 1.25)
Missing	294 (59.2)	590 (61.5)	0.86 (0.63, 1.16)	0.74 (0.49, 1.11)	0.86 (0.62, 1.19)
DCIS nuclear grade					
1	158 (31.8)	294 (30.7)	1^b^	1^b^	1^b^
2	158 (31.8)	338 (35.2)	0.88 (0.67, 1.16)	0.83 (0.59, 1.17)	0.85 (0.64, 1.14)
3	141 (28.4)	277 (28.9)	0.96 (0.71, 1.29)	0.80 (0.55, 1.16)	0.91 (0.66, 1.26)
Missing	40 (8.0)	50 (5.2)	2.20 (1.20, 4.03)	1.01 (0.45, 2.30)	–
DCIS architectural pattern^c,d,e^					
Solid	74 (23.0)	133 (22.2)	1^b^	1^b^	–
Cribriform	103 (32.0)	186 (31.0)	0.97 (0.65, 1.43)	1.29 (0.78, 2.14)	1.04 (0.68, 1.59)
Other	23 (7.1)	39 (6.5)	1.01 (0.56, 1.84)	1.26 (0.59, 2.69)	1.01 (0.54, 1.91)
Mixed	121 (37.6)	242 (40.3)	0.86 (0.59, 1.27)	0.94 (0.58, 1.53)	0.85 (0.57, 1.28)
Missing	1 (0.3)	0 (0)	–	–	–
Necrosis^d^					
None	71 (22.0)	115 (19.2)	1^b^	1^b^	1^b^
Punctate	146 (45.3)	261 (43.5)	0.93 (0.65, 1.33)	1.27 (0.80, 2.04)	0.92 (0.63, 1.35)
Comedo	105 (32.6)	223 (37.2)	0.77 (0.52, 1.14)	0.75 (0.45, 1.25)	0.75 (0.49, 1.13)
Missing^a^	0 (0)	1 (0.1)	–	–	–
Microcalcification^d^					
No	121 (37.6)	202 (33.7)	1^b^	1^b^	1^b^
With DCIS	170 (52.8)	368 (61.3)	0.79 (0.59, 1.05)	0.76 (0.52, 1.10)	0.77 (0.56, 1.06)
Missing	31 (9.6)	30 (5.0)	4.42 (2.31, 8.44)	1.00 (0.06, 17.4)	2.38 (0.19, 29.77)

*OR* odds ratio, *CI* confidence interval

^a^For method of DCIS detection, 1 case was detected by MRI; 2 controls were detected by MRI, 1 by molecular breast imaging, and 3 by ultrasound; for microcalcification, 2 cases and 4 controls had microcalcification in non-neoplastic tissue

^b^Reference category

^c^Includes comedo, micropapillary, papillary, clinging, other (e.g., other features of architectural pattern)

^d^Available for the 322 cases and 600 controls for whom pathology review revealed DCIS

^e^Excluded from the imputation modelling because the model did not converge when this variable was included

**Table 3:** Associations of ER, PR, and HER2 receptor status of DCIS with risk of subsequent invasive breast cancer

Characteristic	Cases (n = 497)	Controls (n = 959)	Univariable OR (95% CI)	Multivariable OR (95% CI)	Multivariable OR (95% CI) with imputation
Estrogen receptor					
Positive (≥ 1% positive)	302 (60.8)	617 (64.3)	1^a^	1^a^	1^a^
Negative	75 (15.1)	146 (15.2)	1.01 (0.74, 1.39)	0.85 (0.57, 1.27)	0.95 (0.69, 1.33)
Missing	120 (24.1)	196 (20.4)	1.80 (1.18, 2.74)	1.37 (0.81, 2.33)	1.71 (1.10, 2.66)
Progesterone receptor					
Positive (≥ 1% positive)	271 (54.5)	537 (56.0)	1^a^	1^a^	1^a^
Negative	104 (20.9)	220 (22.9)	0.90 (0.68, 1.19)	0.78 (0.55, 1.12)	0.86 (0.64, 1.15)
Missing	122 (24.5)	202 (21.1)	1.68 (1.10, 2.57)	1.23 (0.73, 2.09)	1.63 (1.05, 2.53)
HER2					
Negative (0–2+)	265 (53.3)	543 (56.6)	1^a^	1^a^	1^a^
Positive (3+)	52 (10.5)	115 (12.0)	0.93 (0.65, 1.32)	0.80 (0.51, 1.25)	0.88 (0.61, 1.28)
Missing	180 (36.2)	301 (31.4)	2.38 (1.49, 3.82)	2.40 (1.29, 4.47)	2.41 (1.47, 3.97)

*OR* odds ratio, *CI* confidence interval

^a^Reference category

**Table 4:** Associations of clinicopathologic characteristics of DCIS with risk of subsequent ipsilateral invasive breast cancer

Characteristic	Cases (n = 130)	Controls (n = 243)	Multivariable OR (95% CI)
Method of DCIS detection			
Mammogram	108 (83.1)	207 (85.2)	1^a^
Physical exam	14 (10.8)	24 (9.9)	0.68 (0.14, 3.24)
Ultrasound/MRI/Molecular imaging/missing	8 (6.2)	12 (4.9)	0 (0, Inf)
Lesion size (mm)			
< = 8	34 (26.2)	64 (26.3)	1^a^
8–20	41 (31.5)	56 (23.0)	1.81 (0.68, 4.85)
> = 20	42 (32.3)	88 (36.2)	1.48 (0.60, 3.63)
Missing	13 (10.0)	35 (14.4)	1.71 (0.40, 7.26)
DCIS margin			
Clear (≥ 2 mm)	30 (23.1)	56 (23.0)	1^a^
Involved (< 2 mm)	27 (20.8)	42 (17.3)	6.02 (1.25, 29.12)
Missing	73 (56.2)	145 (59.7)	1.46 (0.49, 4.35)
DCIS nuclear grade			
1	42 (32.3)	78 (32.1)	1^a^
2	58 (44.6)	96 (39.5)	0.97 (0.39, 2.46)
3 (high)	30 (23.1)	69 (28.4)	0.33 (0.11, 1.01)
DCIS architectural pattern			
Solid	28 (21.5)	55 (22.6)	1^a^
Cribriform	33 (25.4)	64 (26.3)	2.88 (0.93, 8.97)
Other	13 (10.0)	17 (7.0)	2.77 (0.59, 12.98)
Mixed	56 (43.1)	107 (44.0)	1.19 (0.41, 3.50)
Necrosis			
None	33 (25.4)	37 (15.2)	1^a^
Punctate	56 (43.1)	106 (43.6)	0.84 (0.25, 2.77)
Comedo	41 (31.5)	100 (41.2)	0.17 (0.04, 0.73)
Microcalcification			
No	50 (41.0)	82 (34.9)	1^a^
With DCIS	72 (59.0)	153 (65.1)	0.81 (0.35, 1.84)
(Missing)	8	8	–
Estrogen receptor			
Positive (≥ 1% positive)	94 (79.7)	183 (79.2)	1^a^
Negative	24 (20.3)	48 (20.8)	0.37 (0.13, 1.07)
(Missing)	12	12	–
Progesterone receptor			
Positive (≥ 1% positive)	83 (70.3)	167 (72.3)	1^a^
Negative	35 (29.7)	64 (27.7)	0.43 (0.16, 1.12)
(Missing)	12	12	–
HER2			
Negative (0–2+)	112 (86.2)	201 (82.7)	1^a^
Positive (3+)	18 (13.8)	42 (17.3)	0.71 (0.27, 1.83)

*OR* odds ratio, *CI* confidence interval

^a^Reference category

**Table 5:** Associations of clinicopathologic characteristics of DCIS with risk of subsequent contralateral invasive breast cancer

Characteristic	Cases (n = 162)	Controls (n = 295)	Multivariable OR (95% CI)
Method of DCIS detection			
Mammogram	141 (87.0)	240 (81.4)	1^a^
Physical exam	17 (10.5)	36 (12.2)	0.80 (0.34, 1.87)
Ultrasound/MRI/Molecular imaging/missing^a^	4 (2.5)	19 (6.4)	0.54 (0.09, 3.32)
Lesion size (mm)			
< = 8	42 (25.9)	64 (21.7)	1^a^
8–20	43 (26.5)	108 (36.6)	0.50 (0.24, 1.02)
> = 20	64 (39.5)	96 (32.5)	0.95 (0.44, 2.05)
Missing	13 (8.0)	27 (9.2)	1.06 (0.31, 3.66)
DCIS margin			
Clear (≥ 2 mm)	49 (30.2)	63 (21.4)	1^a^
Involved (< 2 mm)	28 (17.3)	63 (21.4)	0.63 (0.28, 1.43)
Missing	85 (52.5)	169 (57.3)	0.52 (0.23, 1.16)
DCIS nuclear grade			
1	59 (36.4)	116 (39.3)	1^a^
2	54 (33.3)	103 (34.9)	0.90 (0.48, 1.68)
3 (high)	49 (30.2)	76 (25.8)	1.20 (0.63, 2.29)
DCIS architectural pattern			
Solid	40 (24.7)	63 (21.4)	1^a^
Cribriform	54 (33.3)	100 (33.9)	0.72 (0.33, 1.55)
Other	8 (4.9)	16 (5.4)	0.83 (0.22, 3.12)
Mixed	60 (37.0)	116 (39.3)	0.80 (0.40, 1.61)
Necrosis			
None	27 (16.7)	60 (20.4)	1^a^
Punctate	79 (48.8)	129 (43.9)	2.03 (0.94, 4.37)
Comedo	56 (34.6)	105 (35.7)	1.27 (0.59, 2.75)
(Missing)	0	1	–
Microcalcification			
No	58 (38.9)	105 (37.2)	1^a^
With DCIS	91 (61.1)	177 (62.8)	0.91 (0.54, 1.55)
(Missing)	13	13	–
Estrogen receptor			
Positive (≥ 1% positive)	124 (82.1)	221 (82.2)	1^a^
Negative	27 (17.9)	48 (17.8)	1.27 (0.58, 2.78)
(Missing)	11	26	–
Progesterone receptor			
Positive (≥ 1% positive)	116 (76.8)	191 (71.0)	1^a^
Negative	35 (23.2)	78 (29.0)	0.84 (0.42, 1.65)
(Missing)	11	26	–
HER2			
Negative (0–2+)	24(14.8)	45 (15.3)	1^a^
Positive (3+)	138 (85.2)	250 (84.7)	0.99 (0.49, 1.97)

*OR* odds ratio, *CI* confidence interval

^a^Reference category

On performing backward selection starting with a model that included all clinicopathologic variables, only necrosis remained in the model, albeit with statistically non-significant associations with IBC risk overall. Specifically, compared to those with no necrosis, those with punctate necrosis had an OR of 1.25 (0.78–1.99) and those with comedonecrosis had an OR of 0.71 (95% CI 0.43–1.17).

We obtained DCIS tissue for 322 (64.8%) of 497 cases and 600 (62.6%) of 959 controls. There were few differences between those for whom we did and did not obtain DCIS tissue (Supplementary Table 1), although compared to those for whom DCIS tissue was obtained, slightly larger proportions of those for whom DCIS tissue was not obtained had BCS, were White, pre/perimenopausal, and never users of hormone replacement therapy. In sensitivity analyses focused on clinicopathologic variables obtained from both the EMR and from centralized pathology review (i.e., nuclear grade, ER, PR, and HER2), we restricted attention to the results of the centralized pathology review to examine the associations of these variables with IBC risk: for nuclear grade, compared to those with grade 1 DCIS, those with nuclear grade 2 had an OR of 1.08 (95% CI 0.71–1.66) and those with grade 3 had an OR of 0.87 (95% CI 0.55–1.38), while with respect to receptor status, those who were ER negative had an OR of 0.96 (95% CI 0.53–1.73), those who were PR negative had an OR of 0.91 (95% CI 0.53–1.56), and those who were HER2 positive had an OR of 0.80 (95% CI 0.48–1.32).

To examine if the associations of the clinicopathologic variables with risk of IBC varied over time, multivariable conditional logistic regression models with interaction terms between the primary DCIS variables and time to IBC (5–10 years and 10+ years) were fitted. The associations of both punctate and comedo necrosis with risk of IBC diminished over time, most noticeably after 10 years (p < 0.001 and p = 0.012, respectively). Similarly, we found that the association of microcalcification with risk of IBC diminished 10 years after the diagnosis of DCIS (p = 0.001).

We examined the associations of the clinicopathologic variables with IBC risk in several subgroups, including White women (Supplementary Table 2), Black women (Supplementary Table 3), pre-/perimenopausal women (Supplementary Table 4), postmenopausal women (Supplementary Table 5), those who were treated with BCS (Supplementary Table 6), those who received BCS + RT or BCS + RT + HRT (Supplementary Table 7), those who were treated by mastectomy (Supplementary Table 8); furthermore, because the extent of missingness was higher in the earlier part of the DCIS diagnosis eligibility period, we examined IBC risk separately in those whose DCIS was diagnosed either between 1987 and 2001 (Supplementary Table 9) or between 2002 and 2016 (Supplementary Table 10). For the most part, the associations were null, notable exceptions being the strong positive associations with IBC risk in pre/perimenopausal women with punctate necrosis (OR = 5.50, 95% CI 1.61–22.10), in postmenopausal women whose DCIS was detected by physical exam (OR = 1.65, 95% CI 1.01–2.68), and in those treated with BCS who had types of DCIS architectural pattern other than comedo, cribriform, papillary, micropapillary, solid, or clinging (OR = 31.50, 95% CI 2.19–669.80), and the inverse associations with HER2 positivity in those treated with BCS (OR = 0.23, 95% CI 0.04–0.91), with DCIS microcalcification in those who were treated by mastectomy (OR = 0.30, 95% CI 0.12–0.67), and with comedonecrosis (OR = 0.27, 95% CI 0.09–0.70) and microcalcification (OR = 0.35, 95% CI 0.15–0.79) in those with DCIS diagnosed in the earlier part of the eligibility period (1987 to 2001, inclusive). However, most of the associated confidence intervals were very wide.

## Discussion

In the large, diverse, multi-center population-based cohort study reported here, we examined the associations between several clinicopathologic factors (method of DCIS detection, tumor size, DCIS margins status, DCIS grade, comedonecrosis, and ER, PR, and HER2 receptor status) and risk of subsequent invasive breast cancer (IBC) in women with DCIS. For the most part, the associations were null, although some subgroup analyses yielded strong associations. In particular, there were strong positive associations with IBC risk in pre/perimenopausal women with punctate necrosis, in postmenopausal women whose DCIS was detected by physical exam, in those treated with BCS who had DCIS architectural patterns other than the main types, and in those who developed ipsilateral IBC and had involved DCIS margins, and there were inverse associations with HER2 positivity in those treated with BCS and with DCIS microcalcification in those who were treated by mastectomy. However, most of the associated confidence intervals for the latter associations were very wide. The results for the total study population were largely unchanged after reanalysis following imputation for missing variables.

Since the introduction of mammographic screening, there has been a substantial increase in the incidence of DCIS [20], and women with screen-detected DCIS have been observed to have higher risk of subsequent IBC than women in the general population [21]. A recent study showed that women with non-screen detected DCIS also had a higher risk of subsequent IBC than women in the general population, a risk that was higher than that for women with screen-detected DCIS [21]. In the present study, when compared to women whose DCIS was detected by mammogram, we observed that women whose DCIS was detected by physical exam had a small, statistically non-significant increase in the risk of subsequent IBC that was of similar magnitude to that observed in previous studies [10]. However, we did not have detailed information on the reason for detection by physical exam (e.g., self-exam vs. physician exam, etc.).

We did not observe altered risk of IBC overall with either lesion size or margin status. When examined by side of the subsequent IBC, there was a strong positive association (albeit with a wide confidence interval) between involved margins and risk in women who developed an ipsilateral IBC, but no association in those who developed a contralateral IBC. Additionally, there was a statistically non-significant increase in risk of ipsilateral IBC in those with larger DCIS lesions, but not in those who developed contralateral IBC. Previous studies have observed similar findings to ours with respect to risk of ipsilateral IBC [8–11]. A recent study suggested that the association with margin status is confined to those whose DCIS is treated by BCS with or without radiotherapy [11]. Our sample size was insufficient to allow examination of the association by type of treatment for DCIS.

Several other pathologic factors were examined in this study, namely DCIS grade (where high grade is indicative of poorly differentiated lesions), necrosis, architectural (growth) pattern, and microcalcification. None of these variables was associated with risk of subsequent IBC overall or by laterality. However, there were strong positive associations (albeit with wide confidence intervals) between punctate necrosis and IBC risk in pre/perimenopausal women, and in those treated with BCS who had types of DCIS architectural pattern other than comedo, cribriform, papillary, micropapillary, solid, or clinging; in contrast, there was a strong inverse association between microcalcification in DCIS and IBC risk in those who were treated by mastectomy and in those whose DCIS was diagnosed between 1987 and 2001, and in the latter group, there was also an inverse association with comedonecrosis. Numerous previous studies have examined the association between DCIS grade and necrosis with risk of subsequent IBC, and in particular, ipsilateral IBC. Two recent meta-analyses of these associations yielded conflicting findings, reflecting both the heterogeneity of DCIS [22] and inclusion of different studies in these two reports [8, 10]. In the larger and more recent of the two, there was increased risk of ipsilateral IBC in association with high DCIS grade and a suggestion of increased risk with necrosis [8]; the other meta-analysis showed statistically non-significant small increases in risk with grade and with comedonecrosis [10]. There have been only a few studies of IBC risk in association with DCIS architectural pattern and microcalcification [8], rendering conclusions difficult.

While ER, PR, and HER2 are well established prognostic markers for IBC, their utility as prognostic markers for DCIS is less clear [23, 24]. We did not observe associations between the ER, PR, and HER2 receptor status of DCIS and risk of subsequent IBC overall, or in any of the subgroups examined. While earlier reports suggested that risk of subsequent ipsilateral IBC is decreased in women with ER+ and PR+ lesions and increased in those with HER2+ lesions [7, 9], more recent summary estimates of risk presented in meta-analyses suggested no statistically significant alteration in risk in association these markers [8, 10]. In contrast to the present study, many previous studies have been plagued by small sample sizes and relatively short follow-up periods [7–10].

The present study has several strengths. The study population was broadly representative of the racial/ethnic and socioeconomic distribution of the underlying source populations. The study was large, which yielded robust estimates of association that were adjusted for a wide range of potential confounders, including type of DCIS treatment. Furthermore, the large sample size allowed us to undertake analyses in various subgroups of the study population, while the long-term followed up allowed us to examine whether the associations of interest varied over time. In terms of the study limitations, we note that there were missing values for some of the primary variables of interest, which reflects the source of the information, namely, electronic medical records. In particular, there were relatively high missing rates for DCIS margin and receptor status. To address this, we undertook sensitivity analyses after performing multiple imputation and showed that the associations changed little. Also, we were unable to obtain DCIS tissue for some women; however, there were few differences between those for whom we did and did not obtain tissue, so that this is unlikely to have introduced bias into the estimates of association for the tissue-based exposures of interest. Finally, given the many associations that were examined, the few statistically significant associations that we observed may represent chance findings.

In conclusion, with the exception of a few strong associations in specific subgroups, the present study provides little support for associations of the clinicopathological factors studied here with risk of IBC in women with DCIS. Identification of prognostic factors for women with DCIS remains a pressing issue given the potential that this holds for allowing aggressive treatment of DCIS to be limited to those most at risk of progressing to IBC.

## Supplementary Information

Below is the link to the electronic supplementary material.

Supplementary file1 (DOCX 74 KB)

## Data Availability

The datasets generated and/or analyzed during the current study are not publicly available due to patient confidentiality and ongoing use for research purposes but are available from the corresponding author on reasonable request and subject to approval by the IRBs of the participating institutions.
