# Supplementary material for: Clinicopathologic characteristics of ductal carcinoma in situ and risk of subsequent invasive breast cancer: a multicenter, population-based cohort study
Source: Breast Cancer Res Treat. 2025 Jan 20;210(3):615–25. doi: 10.1007/s10549-024-07599-x (PMC11953078; doi:10.1007/s10549-024-07599-x)
Supplement: Supplementary file 1 — Supplementary file1 (DOCX 74 KB) [file 10549_2024_7599_MOESM1_ESM.docx]

**SUPPLEMENTARY INFORMATION**

**Clinicopathologic characteristics of ductal carcinoma in situ and risk of subsequent invasive breast cancer: a multicenter, population-based cohort study**

Thomas E Rohan,^1^ Yihong Wang,^2^ Fergus Couch,^3,4^ Heather Spencer Feigelson,^5^ Robert T. Greenlee,^6^ Stacey Honda,^7^ Azadeh Stark,^8,9^ Dhananjay Chitale,^8,9^ Chenxin Zhang,^1^ Xiaonan Xue,^1^ Mindy Ginsberg,^1^ Olivier Loudig^10^

**Author affiliations**

^1^ Department of Epidemiology and Population Health, Albert Einstein College of Medicine, Bronx, NY, USA

^2^ Department of Pathology and Laboratory Medicine, Rhode Island Hospital and Lifespan Medical Center, Warren Alpert Medical School of Brown University, Providence, RI, USA

^3^ Department of Laboratory Medicine and Pathology, Mayo Clinic, Rochester, MN, USA

^4^ Department of Health Sciences Research, Mayo Clinic, Rochester, MN, USA

^5^ Institute for Health Research, Kaiser Permanente, Aurora, CO, USA

^6^ Center for Clinical Epidemiology and Population Health, Marshfield Clinic Research Institute, Marshfield, WI, USA

^7^ Center for Integrated Healthcare, Kaiser Permanente, Hawaii Permanente Medical Group, Honolulu, HI, USA

^8^ Department of Pathology and Laboratory Medicine, Henry Ford Health System, Detroit, MI, USA

^9^ Breast Oncology Program and Department of Pathology, Henry Ford Health System, Detroit, MI, USA

^10^ Center for Discovery and Innovation (CDI), Hackensack Meridian Health, Nutley, NJ, USA

**CONTENTS**

**Supplementary Table 1.** Baseline characteristics of subjects for whom DCIS tissue was either obtained or not obtained

**Supplementary Table 2.** Associations of clinicopathologic characteristics of DCIS with risk of subsequent invasive breast cancer in White women

**Supplementary Table 3.** Associations of clinicopathologic characteristics of DCIS with risk of subsequent invasive breast cancer in Black women

**Supplementary Table 4.** Associations of clinicopathologic characteristics of DCIS with risk of subsequent invasive breast cancer in pre/perimenopausal women

**Supplementary Table 5.** Associations of clinicopathologic characteristics of DCIS with risk of subsequent invasive breast cancer in postmenopausal women

**Supplementary Table 6.** Associations of clinicopathologic characteristics of DCIS with risk of subsequent invasive breast cancer in women who received breast conserving surgery

**Supplementary Table 7.** Associations of clinicopathologic characteristics of DCIS with risk of subsequent invasive breast cancer in women who received breast conserving surgery

(BCS)+radiotherapy (RT) or CS+RT+hormone therapy

**Supplementary Table 8.** Associations of clinicopathologic characteristics of DCIS with risk of subsequent invasive breast cancer in women who received a mastectomy

**Supplementary Table 9.** Associations of clinicopathologic characteristics of DCIS diagnosed between 1987 and 2001 with risk of subsequent invasive breast cancer

**Supplementary Table 10.** Associations of clinicopathologic characteristics of DCIS diagnosed between 2002 and 2016 with risk of subsequent invasive breast cancer

**Supplementary Table 1.** Baseline characteristics of subjects for whom DCIS tissue was

either obtained or not obtained

| **Variable** | **Tissue obtained**  (n=922) | **Tissue not obtained**  (n= 534) |
| --- | --- | --- |
| Year of DCIS diagnosis |  |  |
| Mean (SD) | 2004.5 (6.4) | 2001.8 (6.7) |
| Median (IQR) | 2005.0 (1999.0, 2010.0) | 2001.0 (1997.0, 2007.0) |
| Treatment |  |  |
| BCS alone | 132 (14.3) | 103 (19.3) |
| BCS + RT | 238 (25.8) | 146 (27.3) |
| BCS + HT and/or RT | 244 (26.5) | 122 (22.8) |
| Mastectomy | 228 (24.7) | 110 (20.6) |
| Missing | 80 (8.7) | 53 (9.9) |
| Race |  |  |
| White | 540 (58.6) | 362 (67.8) |
| Asian | 75 (8.1) | 40 (7.5) |
| Black | 187 (20.3) | 80 (15.0) |
| Mixed/Other | 44 (4.8) | 10 (1.9) |
| Missing | 76 (8.2) | 42 (7.9) |
| Family history of breast cancer |  |  |
| No | 662 (71.8) | 389 (72.8) |
| Yes | 224 (24.3) | 131 (24.5) |
| Missing | 36 (3.9) | 14 (2.6) |
| Age at menarche |  |  |
| < 12 | 104 (11.3) | 45 (8.4) |
| >= 12 | 588 (63.8) | 351 (65.7) |
| Missing | 230 (24.9) | 138 (25.8) |
| Age at first live birth |  |  |
| Never/Not completed pregnancy | 108 (11.7) | 73 (13.7) |
| < 30 | 497 (53.9) | 290 (54.3) |
| >= 30 | 98 (10.6) | 50 (9.4) |
| Missing | 219 (23.8) | 121 (22.7) |
| Bilateral oophorectomy |  |  |
| No | 753 (81.7) | 439 (82.2) |
| Yes | 135 (14.6) | 67 (12.5) |
| Missing | 34 (3.7) | 28 (5.2) |
| Menopausal status |  |  |
| Pre/perimenopausal | 205 (22.2) | 152 (28.5) |
| Postmenopausal | 702 (76.1) | 371 (69.5) |
| Missing | 15 (1.6) | 11 (2.1) |
| Used hormone replacement therapy |  |  |
| Never | 514 (55.7) | 317 (59.4) |
| Former/Current | 315 (34.2) | 161 (30.1) |
| Missing | 93 (10.1) | 56 (10.5) |
| BMI (kg/m2) |  |  |
| < 25 | 248 (26.9) | 150 (28.1) |
| 25 - 29.9 | 279 (30.3) | 159 (29.8) |
| 30-34.9 | 209 (22.7) | 104 (19.5) |
| >=35 | 154 (16.7) | 91 (17.0) |
| Missing | 32 (3.5) | 30 (5.6) |

**Supplementary Table 2.** Associations of clinicopathologic characteristics of DCIS with risk

of subsequent invasive breast cancer in White women

| **Characteristic** | **Cases**  **(n=290)** | **Controls**  **(n= 612)** | **Multivariable**  **OR (95%CI)** |
| --- | --- | --- | --- |
| Method of DCIS detection |  |  |  |
| Mammogram | 240 (82.8) | 529 (86.4) | 1^b^ |
| Physical exam | 32 (11.0) | 56 (9.2) | 1.26 (0.75, 2.10) |
| Ultrasound/MRI/Molecular imaging/missing | 18 (6.2) | 27 (4.4) | 1.44 (0.58, 3.47) |
| Lesion size (mm) |  |  |  |
| <=8 | 99 (34.1) | 165 (27.0) | 1^b^ |
| 8-20 | 82 (28.3) | 185 (30.2) | 0.72 (0.48, 1.06) |
| >=20 | 79 (27.2) | 177 (28.9) | 0.75 (0.49, 1.13) |
| Missing | 30 (10.3) | 85 (13.9) | 0.52 (0.28, 0.92) |
| DCIS margin |  |  |  |
| Clear (≥2mm) | 61 (21.0) | 112 (18.3) | 1^b^ |
| Involved (<2mm) | 36 (12.4) | 90 (14.7) | 0.71 (0.40, 1.25) |
| Missing | 193 (66.6) | 410 (67.0) | 0.75 (0.49, 1.15) |
| DCIS nuclear grade |  |  |  |
| 1 | 91 (31.4) | 176 (28.8) | 1^b^ |
| 2 | 90 (31.0) | 213 (34.8) | 0.83 (0.56, 1.22) |
| 3 (high) | 84 (29.0) | 189 (30.9) | 0.81 (0.54, 1.22) |
| Missing | 25 (8.6) | 34 (5.6) | 1.27 (0.63, 2.53) |
| DCIS architectural pattern^a^ |  |  |  |
| Solid | 38 (21.5) | 82 (22.6) | 1^b^ |
| Cribriform | 58 (32.8) | 115 (31.7) | 1.14 (0.65, 2.01) |
| Other | 10 (5.6) | 27 (7.4) | 0.74 (0.28, 1.82) |
| Mixed | 71 (40.1) | 139 (38.3) | 1.09 (0.63, 1.91) |
| Necrosis^a^ |  |  |  |
| None | 40 (22.6) | 76 (20.9) | 1^b^ |
| Punctate | 80 (45.2) | 147 (40.5) | 1.00 (0.58, 1.73) |
| Comedo | 57 (32.2) | 140 (38.6) | 0.72 (0.41, 1.28) |
| Microcalcification^a^ |  |  |  |
| No | 69 (39.0) | 121 (33.3) | 1^b^ |
| With DCIS | 97 (54.8) | 226 (62.3) | 0.72 (0.47, 1.11) |
| Missing | 11 (6.2) | 16 (4.4) | 1.28 (0.44, 3.61) |
| Estrogen receptor |  |  |  |
| Positive (≥1% positive) | 162 (55.9) | 370 (60.5) | 1^b^ |
| Negative | 42 (14.5) | 91 (14.9) | 1.03 (0.65, 1.62) |
| Missing | 86 (29.7) | 151 (24.7) | 1.20 (0.79, 1.83) |
| Progesterone receptor |  |  |  |
| Positive (≥1% positive) | 146 (50.3) | 325 (53.1) | 1^b^ |
| Negative | 57 (19.7) | 131 (21.4) | 0.91 (0.60, 1.38) |
| Missing | 87 (30.0) | 156 (25.5) | 1.15 (0.75, 1.76) |
| HER2 |  |  |  |
| Negative (0-2+) | 141 (48.6) | 316 (51.6) | 1^b^ |
| Positive (3+) | 30 (10.3) | 82 (13.4) | 0.86 (0.52, 1.40) |
| Missing | 119 (41.0) | 214 (35.0) | 1.22 (0.87, 1.71) |

Abbreviations: OR, odds ratio; CI, confidence interval.

^a^ Available for the 177 cases and 363 controls for whom pathology review revealed DCIS.

^b^ Reference category.**Supplementary Table 3.** Associations of clinicopathologic characteristics of DCIS with risk

of subsequent invasive breast cancer in Black women

| **Characteristic** | **Cases**  **(n=109)** | **Controls**  **(n= 158)** | **Multivariable**  **OR (95%CI)** |
| --- | --- | --- | --- |
| Method of DCIS detection |  |  |  |
| Mammogram | 80 (73.4) | 119 (75.3) | 1^b^ |
| Physical exam | 16 (14.7) | 17 (10.8) | 1.10 (0.40, 3.06) |
| Ultrasound/MRI/Molecular imaging/missing | 13 (11.9) | 22 (13.9) | 0.42 (0.09, 1.59) |
| Lesion size (mm) |  |  |  |
| <=8 | 25 (22.9) | 38 (24.1) | 1^b^ |
| 8-20 | 31 (28.4) | 51 (32.3) | 1.37 (0.54, 3.52) |
| >=20 | 37 (33.9) | 54 (34.2) | 2.00 (0.80, 5.17) |
| Missing | 16 (14.7) | 15 (9.5) | 3.25 (0.76, 14.65) |
| DCIS margin |  |  |  |
| Clear (≥2mm) | 37 (33.9) | 48 (30.4) | 1^b^ |
| Involved (<2mm) | 28 (25.7) | 35 (22.2) | 1.13 (0.48, 2.66) |
| Missing | 44 (40.4) | 75 (47.5) | 0.73 (0.34, 1.58) |
| DCIS nuclear grade |  |  |  |
| 1 | 37 (35.2) | 58 (36.9) | 1^b^ |
| 2 | 32 (30.5) | 60 (38.2) | 0.92 (0.43, 2.00) |
| 3 (high) | 36 (34.3) | 39 (24.8) | 1.70 (0.73, 4.06) |
| Missing | 4 | 1 | - |
| DCIS architectural pattern^a^ |  |  |  |
| Solid | 19 (24.4) | 19 (17.4) | 1^b^ |
| Cribriform | 27 (34.6) | 31 (28.4) | 1.50 (0.51, 4.50) |
| Other | 7 (9.0) | 9 (8.3) | 0.47 (0.10, 2.11) |
| Mixed | 25 (32.1) | 50 (45.9) | 0.45 (0.15, 1.34) |
| Necrosis^a^ |  |  |  |
| None | 19 (24.4) | 19 (17.6) | 1^b^ |
| Punctate | 34 (43.6) | 49 (45.4) | 0.93 (0.32, 2.71) |
| Comedo | 25 (32.1) | 40 (37.0) | 0.75 (0.24, 2.34) |
| Microcalcification^a^ |  |  |  |
| No | 31 (39.7) | 37 (33.9) | 1^b^ |
| With DCIS | 32 (41.0) | 63 (57.8) | 0.55 (0.21, 1.41) |
| Missing | 15 (19.2) | 9 (8.3) | 3.24 (0.80, 14.24) |
| Estrogen receptor |  |  |  |
| Positive (≥1% positive) | 74 (67.9) | 118 (74.7) | 1^b^ |
| Negative | 17 (15.6) | 20 (12.7) | 1.46 (0.54, 3.93) |
| Missing | 18 (16.5) | 20 (12.7) | 0.87 (0.28, 2.61) |
| Progesterone receptor |  |  |  |
| Positive (≥1% positive) | 62 (56.9) | 98 (62.0) | 1^b^ |
| Negative | 29 (26.6) | 39 (24.7) | 0.82 (0.38, 1.73) |
| Missing | 18 (16.5) | 21 (13.3) | 0.62 (0.19, 1.91) |
| HER2 |  |  |  |
| Negative (0-2+) | 66 (60.6) | 103 (65.2) | 1^b^ |
| Positive (3+) | 10 (9.2) | 15 (9.5) | 0.81 (0.25, 2.50) |
| Missing | 33 (30.3) | 40 (25.3) | 1.37 (0.64, 2.93) |

Abbreviations: OR, odds ratio; CI, confidence interval.

^a^ Available for the 78 cases and 109 controls for whom pathology review revealed DCIS.

^b^ Reference category.

**Supplementary Table 4.** Associations of clinicopathologic characteristics of DCIS with risk

of subsequent invasive breast cancer in pre/perimenopausal women

| **Characteristic** | **Cases**  **(n=122)** | **Controls**  **(n= 235)** | **Multivariable**  **OR (95%CI)** |
| --- | --- | --- | --- |
| Method of DCIS detection |  |  |  |
| Mammogram | 94 (77.0) | 192 (81.7) | 1^b^ |
| Physical exam | 17 (13.9) | 35 (14.9) | 0.56 (0.23, 1.27) |
| Ultrasound/MRI/Molecular imaging/missing^a^ | 11 (9.0) | 8 (3.4) | 1.74 (0.41, 7.29) |
| Lesion size (mm) |  |  |  |
| <=8 | 45 (36.9) | 65 (27.7) | 1^b^ |
| 8-20 | 25 (20.5) | 61 (26.0) | 0.64 (0.30, 1.37) |
| >=20 | 39 (32.0) | 66 (28.1) | 1.01 (0.49, 2.10) |
| Missing | 13 (10.7) | 43 (18.3) | 0.40 (0.14, 1.06) |
| DCIS margin |  |  |  |
| Clear (≥2mm) | 31 (25.4) | 41 (17.4) | 1^b^ |
| Involved (<2mm) | 20 (16.4) | 45 (19.1) | 0.79 (0.32, 1.93) |
| Missing | 71 (58.2) | 149 (63.4) | 0.73 (0.36, 1.51) |
| DCIS nuclear grade |  |  |  |
| 1 | 33 (27.0) | 76 (32.3) | 1^b^ |
| 2 | 32 (26.2) | 81 (34.5) | 0.96 (0.47, 1.98) |
| 3 (high) | 43 (35.2) | 67 (28.5) | 1.53 (0.77, 3.07) |
| Missing | 14 (11.5) | 11 (4.7) | 2.62 (0.80, 8.76) |
| DCIS architectural pattern |  |  |  |
| Solid | 20 (27.8) | 28 (21.1) | 1^b^ |
| Cribriform | 21 (29.2) | 36 (27.1) | 1.23 (0.42, 3.68) |
| Other | 5 (6.9) | 7 (5.3) | 0.51 (0.07, 3.19) |
| Mixed | 26 (36.1) | 62 (46.6) | 0.72 (0.26, 1.98) |
| Necrosis |  |  |  |
| None | 15 (20.8) | 23 (17.4) | 1^b^ |
| Punctate | 35 (48.6) | 57 (43.2) | 5.50 (1.61, 22.10) |
| Comedo | 22 (30.6) | 52 (39.4) | 2.66 (0.78,10.28) |
| Missing | 0 | 1 |  |
| Microcalcification |  |  |  |
| No | 26 (36.1) | 47 (35.3) | 1^b^ |
| With DCIS | 40 (55.6) | 81 (60.9) | 1.31 (0.57, 3.11) |
| Missing | 6 (8.3) | 5 (3.8) | 4.52 (0.82, 9.90) |
| Estrogen receptor |  |  |  |
| Positive (≥1% positive) | 43 (35.2) | 97 (41.3) | 1^b^ |
| Negative | 10 (8.2) | 21 (8.9) | 1.21 (0.43, 3.27) |
| Missing | 69 (56.6) | 117 (49.8) | 1.25 (0.69, 2.28) |
| Progesterone receptor |  |  |  |
| Positive (≥1% positive) | 44 (36.1) | 92 (39.1) | 1^b^ |
| Negative | 10 (8.2) | 26 (11.1) | 0.65 (0.23, 1.69) |
| Missing | 68 (55.7) | 117 (49.8) | 1.10 (0.61, 2.00) |
| HER2 |  |  |  |
| Negative (0-2+) | 57 (46.7) | 118 (50.2) | 1^b^ |
| Positive (3+) | 13 (10.7) | 26 (11.1) | 0.81 (0.31, 2.00) |
| Missing | 52 (42.6) | 91 (38.7) | 1.41 (0.78, 2.58) |

Abbreviations: OR, odds ratio; CI, confidence interval.^a^ Available for the 72 cases and 133 controls for whom pathology review revealed DCIS.^b^ Reference category.

**Supplementary Table 5.** Associations of clinicopathologic characteristics of DCIS with risk

of subsequent invasive breast cancer in postmenopausal women

| **Characteristic** | **Cases (n=368)** | **Controls**  **(n=705)** | **Multivariable**  **OR (95%CI)** |
| --- | --- | --- | --- |
| Method of DCIS detection |  |  |  |
| Mammogram | 301 (81.8) | 595 (84.4) | 1^b^ |
| Physical exam | 45 (12.2) | 63 (8.9) | 1.65 (1.01, 2.68) |
| Ultrasound/MRI/Molecular imaging/missing^a^ | 22 (6.0) | 47 (6.7) | 0.87 (0.35, 2.01) |
| Lesion size (mm) |  |  |  |
| <=8 | 104 (28.3) | 185 (26.2) | 1^b^ |
| 8-20 | 118 (32.1) | 225 (31.9) | 0.94 (0.64, 1.39) |
| >=20 | 98 (26.6) | 218 (30.9) | 0.89 (0.59, 1.34) |
| Missing | 48 (13.0) | 77 (10.9) | 1.11 (0.63, 1.95) |
| DCIS margin |  |  |  |
| Clear (≥2mm) | 89 (24.2) | 162 (23.0) | 1^b^ |
| Involved (<2mm) | 59 (16.0) | 111 (15.7) | 1.14 (0.69, 1.88) |
| Missing | 220 (59.8) | 432 (61.3) | 0.85 (0.58, 1.26) |
| DCIS nuclear grade |  |  |  |
| 1 | 122 (33.2) | 216 (30.6) | 1^b^ |
| 2 | 123 (33.4) | 249 (35.3) | 0.79 (0.55, 1.14) |
| 3 (high) | 97 (26.4) | 203 (28.8) | 0.76 (0.51, 1.12) |
| Missing | 26 (7.1) | 37 (5.2) | 0.87 (0.41, 1.80) |
| DCIS architectural pattern^a^ |  |  |  |
| Solid | 53 (21.7) | 101 (22.1) | 1^b^ |
| Cribriform | 80 (32.8) | 148 (32.4) | 1.02 (0.62, 1.68) |
| Other | 17 (7.0) | 30 (6.6) | 0.91 (0.41, 1.97) |
| Mixed | 94 (38.5) | 178 (38.9) | 0.88 (0.54, 1.44) |
| (Missing) | 1 | 0 |  |
| Necrosis^a^ |  |  |  |
| None | 53 (21.6) | 89 (19.5) | 1^b^ |
| Punctate | 111 (45.3) | 200 (43.8) | 0.92 (0.57, 1.51) |
| Comedo | 81 (33.1) | 168 (36.8) | 0.67 (0.40, 1.14) |
| Microcalcification^a^ |  |  |  |
| No | 92 (37.6) | 152 (33.3) | 1^b^ |
| With DCIS | 128 (52.2) | 280 (61.3) | 0.68 (0.45, 1.01) |
| Missing | 25 (10.2) | 25 (5.5) | 1.86 (0.82, 4.27) |
| Estrogen receptor |  |  |  |
| Positive (≥1% positive) | 149 (40.5) | 310 (44.0) | 1^b^ |
| Negative | 39 (10.6) | 82 (11.6) | 1.25 (0.74, 2.08) |
| Missing | 180 (48.9) | 313 (44.4) | 1.15 (0.81, 1.62) |
| Progesterone receptor |  |  |  |
| Positive (≥1% positive) | 129 (35.1) | 265 (37.6) | 1^b^ |
| Negative | 59 (16.0) | 127 (18.0) | 1.04 (0.66, 1.64) |
| Missing | 180 (48.9) | 313 (44.4) | 1.09 (0.76, 1.56) |
| HER2 |  |  |  |
| Negative (0-2+) | 203 (55.2) | 418 (59.3) | 1^b^ |
| Positive (3+) | 39 (10.6) | 86 (12.2) | 1.01 (0.62, 1.63) |
| Missing | 126 (34.2) | 201 (28.5) | 1.17 (0.84, 1.64) |

Abbreviations: OR, odds ratio; CI, confidence interval.

^a^ Available for the 245 cases and 457 controls for whom pathology review revealed DCIS.

^b^ Reference category.

**Supplementary Table 6.** Associations of clinicopathologic characteristics of DCIS with risk of

subsequent invasive breast cancer in women who received breast conserving surgery

| **Characteristic** | **Cases**  **(n=97)** | **Controls**  **(n= 138)** | **Multivariable**  **OR (95%CI)** |
| --- | --- | --- | --- |
| Method of DCIS detection |  |  |  |
| Mammogram | 72 (74.2) | 121 (87.7) | 1^b^ |
| Physical exam | 17 (17.5) | 14 (10.1) | 2.15 (0.79, 5.95) |
| Ultrasound/MRI/Molecular imaging/missing^a^ | 8 (8.2) | 3 (2.2) | 8.73 (1.52, 72.48) |
| Lesion size (mm) |  |  |  |
| <=8 | 40 (41.2) | 57 (41.3) | 1^b^ |
| 8-20 | 30 (30.9) | 40 (29.0) | 1.30 (0.59, 2.89) |
| >=20 | 18 (18.6) | 27 (19.6) | 1.23 (0.50, 3.03) |
| Missing | 9 (9.3) | 14 (10.1) | 1.23 (0.27, 5.06) |
| DCIS margin |  |  |  |
| Clear (≥2mm) | 21 (21.6) | 26 (18.8) | 1^b^ |
| Involved (<2mm) | 14 (14.4) | 14 (10.1) | 1.46 (0.40, 5.37) |
| Missing | 62 (63.9) | 98 (71.0) | 1.0 (0.38, 2.66) |
| DCIS nuclear grade |  |  |  |
| 1 | 37 (38.1) | 47 (34.1) | 1^b^ |
| 2 | 35 (36.1) | 50 (36.2) | 0.66 (0.29, 1.48) |
| 3 (high) | 18 (18.6) | 26 (18.8) | 0.69 (0.25, 1.83) |
| Missing | 7 (7.2) | 15 (10.9) | 0.64 (0.15, 2.41) |
| DCIS architectural pattern^a^ |  |  |  |
| Solid | 14 (25.0) | 22 (29.3) | 1^b^ |
| Cribriform | 15 (26.8) | 21 (28.0) | 2.84 (0.66, 14.06) |
| Other | 5 (8.9) | 5 (6.7) | 31.50 (2.19, 669.80) |
| Mixed | 22 (39.3) | 27 (36.0) | 2.50 (0.65, 10.35) |
| (Missing) | 1 | 0 | - |
| Necrosis^a^ |  |  |  |
| None | 20 (35.1) | 25 (33.3) | 1^b^ |
| Punctate | 25 (43.9) | 28 (37.3) | 0.73 (0.22, 2.42) |
| Comedo | 12 (21.1) | 22 (29.3) | 0.31 (0.06, 1.46) |
| Microcalcification^a^ |  |  |  |
| No | 26 (45.6) | 31 (41.3) | 1^b^ |
| With DCIS | 22 (38.6) | 39 (52.0) | 0.57 (0.18, 1.74) |
| Missing | 9 (15.8) | 5 (6.7) | 2.99 (0.49, 21.69) |
| Estrogen receptor |  |  |  |
| Positive (≥1% positive) | 31 (32.0) | 45 (32.6) | 1^b^ |
| Negative | 7 (7.2) | 9 (6.5) | 0.67 (0.13, 3.18) |
| Missing | 59 (60.8) | 84 (60.9) | 1.93 (0.80, 4.82) |
| Progesterone receptor |  |  |  |
| Positive (≥1% positive) | 31 (32.0) | 44 (31.9) | 1^b^ |
| Negative | 6 (6.2) | 10 (7.2) | 0.75 (0.15, 3.35) |
| Missing | 60 (61.9) | 84 (60.9) | 1.97 (0.82, 4.91) |
| HER2 |  |  |  |
| Negative (0-2+) | 50 (51.5) | 71 (51.4) | 1^b^ |
| Positive (3+) | 3 (3.1) | 18 (13.0) | 0.23 (0.04, 0.91) |
| Missing | 44 (45.4) | 49 (35.5) | 1.59 (0.76, 3.35) |

Abbreviations: OR, odds ratio; CI, confidence interval.

^a^ Available for the 57 cases and 75 controls for whom pathology review revealed DCIS.

^b^ Reference category.

**Supplementary Table 7.** Associations of clinicopathologic characteristics of DCIS with risk

of subsequent invasive breast cancer in women who received breast conserving surgery

(BCS)+radiotherapy (RT) or CS+RT+hormone therapy

| **Characteristic** | **Cases (n=228)** | **Controls**  **(n= 454)** | **Multivariable**  **OR (95%CI)** |
| --- | --- | --- | --- |
| Method of DCIS detection |  |  |  |
| Mammogram | 195 (85.5) | 394 (86.8) | 1^b^ |
| Physical exam | 21 (9.2) | 38 (8.4) | 0.95 (0.48, 1.80) |
| Ultrasound/MRI/Molecular imaging/missing^a^ | 12 (5.3) | 22 (4.8) | 0.39 (0.08, 1.30) |
| Lesion size (mm) |  |  |  |
| <=8 | 77 (33.8) | 116 (25.6) | 1^b^ |
| 8-20 | 76 (33.3) | 166 (36.6) | 0.58 (0.37, 0.91) |
| >=20 | 52 (22.8) | 127 (28.0) | 0.61 (0.38, 1.00) |
| Missing | 23 (10.1) | 45 (9.9) | 0.57 (0.29, 1.10) |
| DCIS margin |  |  |  |
| Clear (≥2mm) | 59 (25.9) | 122 (26.9) | 1^b^ |
| Involved (<2mm) | 49 (21.5) | 98 (21.6) | 1.20 (0.70, 2.06) |
| Missing | 120 (52.6) | 234 (51.5) | 1.00 (0.64, 1.57) |
| DCIS nuclear grade |  |  |  |
| 1 | 76 (33.3) | 131 (28.9) | 1^b^ |
| 2 | 69 (30.3) | 168 (37.0) | 0.68 (0.43, 1.06) |
| 3 (high) | 71 (31.1) | 140 (30.8) | 0.90 (0.57, 1.41) |
| Missing | 12 (5.3) | 15 (3.3) | 0.97 (0.36, 2.55) |
| DCIS architectural pattern^a^ |  |  |  |
| Solid | 36 (23.7) | 72 (24.7) | 1^b^ |
| Cribriform | 56 (36.8) | 83 (28.4) | 1.32 (0.73, 2.42) |
| Other | 9 (5.9) | 20 (6.8) | 0.72 (0.26, 1.88) |
| Mixed | 51 (33.6) | 117 (40.1) | 0.84 (0.46, 1.52) |
| Necrosis^a^ |  |  |  |
| None | 25 (16.4) | 49 (16.8) | 1^b^ |
| Punctate | 75 (49.3) | 136 (46.7) | 1.42 (0.75, 2.75) |
| Comedo | 52 (34.2) | 106 (36.4) | 1.04 (0.52, 2.10) |
| (Missing) | 0 | 1 |  |
| Microcalcification^a^ |  |  |  |
| No | 53 (34.9) | 97 (33.2) | 1^b^ |
| With DCIS | 87 (57.2) | 180 (61.6) | 0.99 (0.61, 1.60) |
| Missing | 12 (7.9) | 15 (5.1) | 0.90 (0.30, 2.52) |
| Estrogen receptor |  |  |  |
| Positive (≥1% positive) | 94 (41.2) | 205 (45.2) | 1^b^ |
| Negative | 29 (12.7) | 58 (12.8) | 1.39 (0.77, 2.48) |
| Missing | 105 (46.1) | 191 (42.1) | 1.21 (0.81, 1.82) |
| Progesterone receptor |  |  |  |
| Positive (≥1% positive) | 84 (36.8) | 176 (38.8) | 1^b^ |
| Negative | 40 (17.5) | 87 (19.2) | 1.00 (0.59, 1.70) |
| Missing | 104 (45.6) | 191 (42.1) | 1.08 (0.72, 1.63) |
| HER2 |  |  |  |
| Negative (0-2+) | 132 (57.9) | 278 (61.2) | 1^b^ |
| Positive (3+) | 21 (9.2) | 41 (9.0) | 1.24 (0.64, 2.37) |
| Missing | 75 (32.9) | 135 (29.7) | 1.13 (0.75, 1.70) |

Abbreviations: OR, odds ratio; CI, confidence interval.

^a^ Available for the 152 cases and 292 controls for whom pathology review revealed DCIS.

^b^ Reference category.

**Supplementary Table 8.** Associations of clinicopathologic characteristics of DCIS with risk

of subsequent invasive breast cancer in women who received a mastectomy

| **Characteristic** | **Cases (n=101)** | **Controls**  **(n= 237)** | **Multivariable**  **OR (95%CI)** |
| --- | --- | --- | --- |
| Method of DCIS detection |  |  |  |
| Mammogram | 75 (74.3) | 189 (79.7) | 1^b^ |
| Physical exam | 19 (18.8) | 39 (16.5) | 1.34 (0.65, 2.73) |
| Ultrasound/MRI/Molecular imaging/missing^a^ | 7 (6.9) | 9 (3.8) | 3.79 (0.67, 23.14) |
| Lesion size (mm) |  |  |  |
| <=8 | 13 (12.9) | 35 (14.8) | 1^b^ |
| 8-20 | 20 (19.8) | 50 (21.1) | 0.87 (0.32, 2.36) |
| >=20 | 54 (53.5) | 117 (49.4) | 1.38 (0.61, 3.30) |
| Missing | 14 (13.9) | 35 (14.8) | 0.82 (0.27, 2.47) |
| DCIS margin |  |  |  |
| Clear (≥2mm) | 18 (17.8) | 28 (11.8) | 1^b^ |
| Involved (<2mm) | 10 (9.9) | 24 (10.1) | 0.88 (0.23, 3.13) |
| Missing | 73 (72.3) | 185 (78.1) | 0.64 (0.27, 1.53) |
| DCIS nuclear grade |  |  |  |
| 1 | 21 (20.8) | 61 (25.7) | 1^b^ |
| 2 | 33 (32.7) | 78 (32.9) | 1.20 (0.56, 2.59) |
| 3 (high) | 37 (36.6) | 84 (35.4) | 1.21 (0.58, 2.55) |
| Missing | 10 (9.9) | 14 (5.9) | 2.84 (0.78, 10.55) |
| DCIS architectural pattern^a^ |  |  |  |
| Solid | 16 (22.9) | 26 (16.5) | 1^b^ |
| Cribriform | 19 (27.1) | 52 (32.9) | 0.72 (0.26, 1.96) |
| Other | 5 (7.1) | 10 (6.3) | 1.54 (0.31, 7.68) |
| Mixed | 30 (42.9) | 70 (44.3) | 0.71 (0.27, 1.89) |
| Necrosis^a^ |  |  |  |
| None | 9 (12.9) | 21 (13.3) | 1^b^ |
| Punctate | 30 (42.9) | 56 (35.4) | 0.97 (0.33, 2.87) |
| Comedo | 31 (44.3) | 81 (51.3) | 0.51 (0.18, 1.43) |
| Microcalcification^a^ |  |  |  |
| No | 23 (32.9) | 42 (26.6) | 1^b^ |
| With DCIS | 42 (60.0) | 113 (71.5) | 0.30 (0.12, 0.67) |
| Missing | 5 (7.1) | 3 (1.9) | 4.87 (0.62, 51.34) |
| Estrogen receptor |  |  |  |
| Positive (≥1% positive) | 35 (34.7) | 100 (42.2) | 1^b^ |
| Negative | 11 (10.9) | 29 (12.2) | 0.97 (0.36, 2.45) |
| Missing | 55 (54.5) | 108 (45.6) | 1.41 (0.74, 2.73) |
| Progesterone receptor |  |  |  |
| Positive (≥1% positive) | 29 (28.7) | 85 (35.9) | 1^b^ |
| Negative | 17 (16.8) | 44 (18.6) | 1.14 (0.49, 2.63) |
| Missing | 55 (54.5) | 108 (45.6) | 1.50 (0.76, 3.01) |
| HER2 |  |  |  |
| Negative (0-2+) | 45 (44.6) | 126 (53.2) | 1^b^ |
| Positive (3+) | 23 (22.8) | 46 (19.4) | 1.26 (0.60, 2.60) |
| Missing | 33 (32.7) | 65 (27.4) | 1.20 (0.61, 2.33) |

Abbreviations: OR, odds ratio; CI, confidence interval.

^a^ Available for the 70 cases and 158 controls for whom pathology review revealed DCIS.

^b^ Reference category.

**Supplementary Table 9.** Associations of clinicopathologic characteristics of DCIS diagnosed

between 1987 and 2001 with risk of subsequent invasive breast cancer

| **Characteristic** | **Cases**  **(n=200)** | **Controls**  **(n= 397)** | **Multivariable**  **OR (95%CI)** |
| --- | --- | --- | --- |
| Method of DCIS detection |  |  |  |
| Mammogram | 156 (78.0) | 317 (79.8) | 1^b^ |
| Physical exam | 27 (13.5) | 53 (13.4) | 0.76 (0.38, 1.55) |
| Ultrasound/MRI/Molecular imaging/missing | 17 (8.5) | 27 (6.8) | 0.95 (0.23, 3.90) |
| Lesion Size (mm) |  |  |  |
| <=8 | 64 (32.0) | 107 (27.0) | 1^b^ |
| 8-20 | 49 (24.5) | 108 (27.2) | 0.62 (0.33, 1.17) |
| >=20 | 54 (27.0) | 116 (29.2) | 0.75 (0.40, 1.41) |
| Missing | 33 (16.5) | 66 (16.6) | 1.17 (0.42, 3.26) |
| DCIS margin |  |  |  |
| Clear (≥2mm) | 27 (13.5) | 40 (10.1) | 1^b^ |
| Involved (<2mm) | 30 (15.0) | 48 (12.1) | 1.21 (0.42, 3.49) |
| Missing | 143 (71.5) | 309 (77.8) | 0.56 (0.23, 1.37) |
| DCIS nuclear grade |  |  |  |
| 1 | 66 (33.0) | 108 (27.2) | 1^b^ |
| 2 | 51 (25.5) | 130 (32.7) | 0.72 (0.38, 1.38) |
| 3 | 49 (24.5) | 112 (28.2) | 0.62 (0.31, 1.23) |
| Missing | 34 (17.0) | 47 (11.8) | 0.89 (0.30, 2.61) |
| DCIS architectural pattern^a^ |  |  |  |
| Solid | 29 (26.4) | 49 (23.7) | 1^b^ |
| Cribriform | 30 (27.3) | 65 (31.4) | 1.40 (0.48, 4.13) |
| Other | 10 (9.1) | 17 (8.2) | 1.26 (0.28, 5.68) |
| Mixed | 41 (37.3) | 76 (36.7) | 1.21 (0.46, 3.15) |
| Necrosis^a^ |  |  |  |
| None | 31 (28.2) | 33 (15.9) | 1^b^ |
| Punctate | 49 (44.5) | 87 (42.0) | 0.63 (0.24, 1.69) |
| Comedo | 30 (27.3) | 87 (42.0) | 0.27 (0.09, 0.79) |
| Microcalcification^a^ |  |  |  |
| No | 51 (46.4) | 66 (31.9) | 1^b^ |
| With DCIS | 52 (47.3) | 136 (65.7) | 0.35 (0.15, 0.79) |
| Missing | 7 (6.4) | 5 (2.4) | -^c^ |
| Estrogen receptor |  |  |  |
| Positive (≥1% positive) | 82 (41.0) | 181 (45.6) | 1^b^ |
| Negative | 23 (11.5) | 43 (10.8) | 0.96 (0.42, 2.21) |
| Missing | 95 (47.5) | 173 (43.6) | 1.43 (0.68, 3.01) |
| Progesterone receptor |  |  |  |
| Positive (≥1% positive) | 77 (38.5) | 161 (40.6) | 1^b^ |
| Negative | 27 (13.5) | 59 (14.9) | 0.79 (0.36, 1.73) |
| Missing | 96 (48.0) | 177 (44.6) | 1.45 (0.69, 3.06) |
| HER2/neu |  |  |  |
| Negative (0-2+) | 88 (44.0) | 191 (48.1) | 1^b^ |
| Positive (3+) | 19 (9.5) | 48 (12.1) | 0.64 (0.28, 1.46) |
| Missing | 93 (46.5) | 158 (39.8) | 4.92 (1.28, 18.94) |

^a^ Available for the 110 cases and 207 controls for whom pathology review revealed DCIS.

^b^ Reference category.

^c^ The missing group was excluded from the model due to its small size.

**Supplementary Table 10.** Associations of clinicopathologic characteristics of DCIS diagnosed

between 2002 and 2016 with risk of subsequent invasive breast cancer

| **Characteristic** | **Cases**  **(n=297)** | **Controls**  **(n= 562)** | **Multivariable**  **OR (95%CI)** |
| --- | --- | --- | --- |
| Method of DCIS detection |  |  |  |
| Mammogram | 242 (81.5) | 484 (86.1) | 1^b^ |
| Physical exam | 36 (12.1) | 47 (8.4) | 1.77 (0.90, 3.46) |
| Ultrasound/MRI/Molecular imaging/missing | 19 (6.4) | 31 (5.5) | 1.79 (0.53, 6.07) |
| Lesion Size (mm) |  |  |  |
| <=8 | 88 (29.6) | 149 (26.5) | 1^b^ |
| 8-20 | 96 (32.3) | 182 (32.4) | 0.95 (0.58, 1.55) |
| >=20 | 84 (28.3) | 172 (30.6) | 0.88 (0.53, 1.45) |
| Missing | 29 (9.8) | 59 (10.5) | 0.68 (0.30, 1.57) |
| DCIS margin |  |  |  |
| Clear (≥2mm) | 94 (31.6) | 166 (29.5) | 1^b^ |
| Involved (<2mm) | 52 (17.5) | 115 (20.5) | 0.90 (0.51, 1.57) |
| Missing | 151 (50.8) | 281 (50.0) | 0.83 (0.49, 1.41) |
| DCIS nuclear grade |  |  |  |
| 1 | 92 (31.0) | 186 (33.1) | 1^b^ |
| 2 | 107 (36.0) | 208 (37.0) | 0.91 (0.58, 1.42) |
| 3 | 92 (31.0) | 165 (29.4) | 0.99 (0.61, 1.60) |
| Missing | 6 (2.0) | 3 (0.5) | 0.94 (0.12, 7.14) |
| DCIS architectural pattern^a^ |  |  |  |
| Solid | 45 (21.2) | 84 (21.4) | 1^b^ |
| Cribriform | 73 (34.4) | 121 (30.8) | 1.12 (0.58, 2.14) |
| Other | 13 (6.1) | 22 (5.6) | 1.38 (0.51, 3.72) |
| Mixed | 80 (37.7) | 166 (42.2) | 0.68 (0.35, 1.31) |
| Missing | 1 (0.5) | 0 (0.0) | - ^c^ |
| Necrosis^a^ |  |  |  |
| None | 40 (18.9) | 82 (20.9) | 1^b^ |
| Punctate | 97 (45.8) | 174 (44.3) | 1.71 (0.92, 3.19) |
| Comedo | 75 (35.4) | 136 (34.6) | 1.14 (0.60, 2.16) |
| Missing | 0 (0.0) | 1 (0.3) | - ^c^ |
| Microcalcification^a^ |  |  |  |
| No | 70 (33.0) | 136 (34.6) | 1^b^ |
| With DCIS | 118 (55.7) | 232 (59.0) | 1.06 (0.65, 1.74) |
| Missing | 24 (11.3) | 25 (6.4) | 1.46 (0.08, 26.71) |
| Estrogen receptor |  |  |  |
| Positive (≥1% positive) | 220 (74.1) | 436 (77.6) | 1^b^ |
| Negative | 52 (17.5) | 103 (18.3) | 0.84 (0.50, 1.40) |
| Missing | 25 (8.4) | 23 (4.1) | 1.37 (0.58, 3.22) |
| Progesterone receptor |  |  |  |
| Positive (≥1% positive) | 194 (65.3) | 376 (66.9) | 1^b^ |
| Negative | 77 (25.9) | 161 (28.6) | 0.79 (0.51, 1.25) |
| Missing | 26 (8.8) | 25 (4.4) | 1.04 (0.44, 2.47) |
| HER2/neu |  |  |  |
| Negative (0-2+) | 177 (59.6) | 352 (62.6) | 1^b^ |
| Positive (3+) | 33 (11.1) | 67 (11.9) | 0.95 (0.52, 1.70) |
| Missing | 87 (29.3) | 143 (25.4) | 1.85 (0.77, 4.47) |

^a^ Available for the 212 cases and 393 controls for whom pathology review revealed DCIS.

^b^ Reference category.

^c^ The missing group was excluded from the model due to the small sample size.
